# Recurrent Incisional Hernia due to Pseudomyxoma Peritonei

**DOI:** 10.1155/2011/853906

**Published:** 2011-05-18

**Authors:** Zafer Ergul, Engin Olcucuoglu, Hakan Kulacoglu, Cenap Dener

**Affiliations:** ^1^Department of Surgery, Diskapi Yildirim Beyazit Teaching and Research Hospital, Irfan Bastug Caddesi, 06110 Ankara, Turkey; ^2^Department of Surgery, Faculty of Medicine, Fatih University, Alparslan Turkes Caddesi no. 57, Emek, 06510 Ankara, Turkey

## Abstract

Pseudomyxoma peritonei is a rare but challenging neoplastic disease which is characterized with intraperitoneal mucinous-gelatinous fluid accumulation. It rarely presents as a mass mimicking abdominal wall hernias A recurrent incisional hernia due to pseudomyxoma peritonei is presented here. A 60-year-old female patient had been operated on for a left mucinous ovarian cyst 20 cm in diameter in 1998. Mucinous material had disseminated into interloop spaces through the right subdiaphragmatic region. Total abdominal hysterectomy + bilateral salpingooophorectomy and peritoneal toilet had been performed. She was rehospitalized for abdominal distention and a 4 cm defect over the incision and underwent a hernia repair using polypropylene mesh in 2001. Abdominal distention recurred to give a rise to an incisional hernia in 2006. She was reoperated for decompression and repair, but nothing could be done because of sticky adhesions and the incision were simply closed. The patient was referred to our department for operation. A prosthetic hernia repair with 30 × 30 cm polypropylene mesh was performed. The patient was discharged on the postoperative 5th day following an uneventful recovery. However, she died of disseminated disease after 18 months.

## 1. Introduction

Pseudomyxoma peritonei is a rare but challenging neoplastic disease. It is characterized with intraperitoneal mucinous-gelatinous fluid accumulation. The origin is mostly mucinous cysts of the ovary or appendix vermiformis [1–3]. Pseudomyxoma peritonei rarely presents as a mass mimicking abdominal wall hernias [4–8]. The reports describing the relation between pseudomyxoma peritonei and incisional hernia are less common [9]. A recurrent incisional hernia due to pseudomyxoma peritonei is presented here.

## 2. Case Presentation

**Figure 1:**
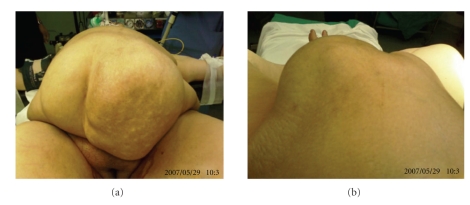
Patient on the operation table. (a) Large incisional hernia seen from the patient's leg side. (b) The view of the hernia from the cranial side.

**Figure 2:**
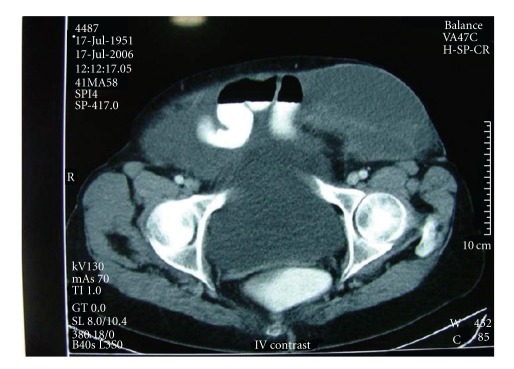
Magnetic resonance imaging of the case (IV contrast, T1). A large incisional hernia containing bowel loops and a great amount of fluid.

**Figure 3:**
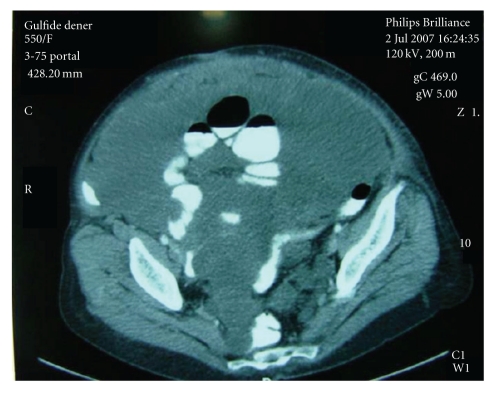
Computed tomographic features of the case. A very large incisional hernia is seen. Bowel loops float within mucinous ascites.

A 60-year-old female patient had been operated on for a left mucinous ovarian cyst 20 cm in diameter in another center in February 1998. Mucinous material had disseminated into interloop spaces through the right subdiaphragmatic region. Total abdominal hysterectomy + bilateral salpingooophorectomy and peritoneal toilet had been performed. She was rehospitalized for abdominal distention and underwent surgery with diagnosis of pseudomyxoma peritonei in December 2001. A 4 cm defect over the incision was observed, and a hernia repair using polypropylene mesh was performed. Abdominal distention recurred by time and gave a rise to an incisional hernia in Pfannenstiel incision in May 2004. She was operated on again for decompression and repair, but the operating team could not achieve this intent because of sticky adhesions and simply closed the incision. As the hernia became gigantic to limit the patient's daily life within the next 2 years, the patient was referred to our department for operation. Her abdominal girth remarkably enlarged. A very large hernia, mostly located on the left side, existed (Figure 1). MRI dated 2006 displayed a large incisional hernia including bowel loops, and the abdomen was full of massive intra-abdominal mucinous material deposition (Figure 2). She denied a reoperation first; however her complaint got worse and a CT dated 2006 displayed a further increase of herniation (Figure 3). She then accepted a surgical intervention. In the operation, a meshoma was found. Abdomen was full of a large amount of mucinous fluid and gelatinous material. Following abdominal decompression the bowel loops put back into the abdominal cavity. A prosthetic hernia repair with an onlay 30 × 30 cm polypropylene mesh was performed. The patient was discharged on the postoperative 5th day following an uneventful recovery. Although abdominal distention has become a problem again after the operation she is doing well at the postoperative 9th month with no sign of recurrence in physical examination and no fascial defect on ultrasound. Nevertheless it was known by phone call that she died of disseminated disease 18 month after the operation.

## 3. Discussion

The prognosis of pseudomyxoma peritonei is poor. Five-year survival rate is less than 50% [1, 10]. Recently heated intraperitoneal chemotherapy has been reported to provide 62.5% to 100% survival rates for low-grade and 0%–65% rates for high-grade disease [1]. 

Patients with pseudomyxoma peritonei usually complain with an increase in abdominal girth [2]. However, an obvious abdominal wall hernia is not common. There are a limited number of case reports in the literature describing an inguinal [3–7], femoral [9], or umbilical [11] hernia secondary to pseudomyxoma peritonei. Herniation may be a result of recurrence of a previously diagnosed pseudomyxoma peritonei [7]. On the other hand, mucinous material may be found in hernia sac without any previous specific diagnosis [3]. 

Abdominal pain is the most common complaint of pseudomyxoma peritonei with an incidence of 23% in the initial evaluation, whereas a newly onset hernia is seen in 12% of the cases [2]. A report from Washington Cancer Institute revealed that 14% of the patients with pseudomyxoma peritonei first presented with a new onset hernia, of which the majority were inguinal hernias [1]. Increased intraabdominal pressure due to gradual accumulation of mucinous ascites is the cause of herniation. This high pressure forced the laparotomy incision, and intestinal loops may be herniated with an amount of fluid. A recurrent incisional hernia on the previous incision after primary repair has not been reported to date, whereas a case of incisional hernia was reported in an Italian more than 50 years ago [9]. In fact, ideal treatment for pseudomyxoma peritonei is under debate. Patients with abdominal wall hernias due to pseudomyxoma peritonei are possibly best treated with prosthetic repairs.

## 4. Conclusions

Pseudomyxoma peritonei is a very rare cause of abdominal wall hernias. The results of surgical interventions are not promising, but remain the only option for the patient with poor quality of life.

##  Authors' Contribution 

Z. Ergul, E. Olcucuoglu, and H. Kulacoglu joined the second hernia repair. Z. Ergul, E. Olcucuoglu, and H. Kulacoglu did the literature review. H. Kulacoglu wrote the paper. C. Dener performed the first hernia repair. C. Dener got the imaging studies done.

##  Conflict of Interests 

The authors declare no conflict of interests.
